# Ten simple rules for successfully hosting an intern at a scientific software company

**DOI:** 10.1371/journal.pcbi.1007020

**Published:** 2019-06-20

**Authors:** Kristine Briedis, Yi-Shiou Chen, Scott Markel

**Affiliations:** Dassault Systèmes BIOVIA, San Diego, California, United States of America; Whitehead Institute for Biomedical Research, UNITED STATES

Internships are increasingly popular choices for motivated students looking to enhance their skills, explore various employers, and get a head start on finding full-time employment. Although there are a few Ten Simple Rules articles from the intern’s [1,2] and mentor’s [3] perspectives, there are none written from the employer’s. We’d like to share what we’ve learned over the years.

We work for a scientific software company, focusing on writing data pipelining software for our computational biology customers, primarily in pharmaceutical and biotech companies. Although we are informed by our specific experience, many of the following rules could be applied to internships in other fields, as well as academia. In general, we believe that these rules also apply to startup companies. The biggest difference one of us has experienced is that there may not be the luxury of having enough time to spend preparing tasks and working with interns. And all tasks may be on the critical path.

## Rule 1: Interview well

Start early. Most of the find-an-intern process is now taking place in the fall for summer internships. When seeking an intern, we must first recognize that, especially for undergraduates, we are unlikely to find exactly the background we desire. Most students will have either a computer science major or are studying biology. Some may be in bioinformatics programs, but in our experience, this is more likely an option for master’s degree students. We also need to decide if we prefer undergraduates or graduate students. One aspect is the pay rate. Another is that graduate students are already starting to specialize. Clearly describe what the intern will work on and accomplish during the internship. Let them know whether any of the work will be publishable.

Where to find candidates? We attend career fairs and post openings online. We talk with previous interns, the schools and programs in which they studied, and colleagues and friends both in our company and in the community. Leverage your professional networks. If your academic contacts invite you to present to their classes or student groups, say yes.

This is in addition to the standard hiring and tracking pipeline in place at our company. Dassault Systèmes has a well-monitored approach to interns. Descriptions are posted online, and our human resources (HR) department attends career fairs nationwide. The company also maintains a database of past applicants that can be mined for promising candidates. We track by brand, e.g., BIOVIA, and geography. We are interested in knowing interns sought, interns hired, interns started, interns who successfully complete, interns hired by the groups where they interned, interns who were hired within the same brand but a different group, and interns hired by different brands. This information has been gathered for more than 15 years within the larger company. A small company, e.g., a startup, might not have the history or infrastructure to support this, although the same information is likely to exist informally.

The interview process may be stressful for the students. Many have not previously interviewed at companies. Keep in mind that undergraduates will probably not have the same skills one would look for in a new hire for a full-time position. Instead, they’re still learning.

## Rule 2: Plan for arrival

Internships often have a very short duration, sometimes only 2 or 3 summer months. You and your intern have to be ready to start on the first day. They’ll typically be looking to you, as this is often very new to them.

Know what you want them to learn or become familiar with during those first days and weeks. Choose projects in advance (see Rule 5). Think about having your intern sit near team mentors. It makes answering questions more convenient for both of you. Talk with others who have had interns, especially at your own company. See what suggestions they have, including what didn’t work so well. You may also need to provide the usual company overview for your intern; HR may not provide the same kind of new-hire orientation, or any at all, for interns.

## Rule 3: Introduce environment and tools

Many interns have not developed software in a commercial environment. They may not be familiar with common tools for bug and feature tracking, source code control, continuous build/integration, etc. Providing short tutorials and online help will enable your intern to be productive faster. They’ll learn best by doing. You might want to provide a sandbox environment or make sure interns are appropriately monitored, especially when it comes to code check-ins.

Another aspect of a commercial environment is the commercial part. This may be new to the interns, as they may not have interacted much with private industry. They may need to sign nondisclosure agreements (NDAs), which is almost always a first for them. Another point of caution is customer meetings/calls. The shift from a more open academic environment to a commercial one often entails being a bit more circumspect in conversations. Interns typically need to be coached on this if you wish to include them in customer meetings. Alternatively, you might decide it is best to leave them out of confidential conversations.

## Rule 4: Mentor commercial software development skills

Provide a guided tour of your organization’s best practices, including agile methods, if applicable. Our company has a good training checklist for newly hired software engineers provided by the quality management system team that we also use for interns. If possible, having an intern start at the same time as a new permanent employee allows for some reduction in needing to cover introductory material about the company, processes, tools, and tasks. It is critical for them to develop a good working knowledge of your source code control and issue tracking systems. Introduce task planning and code reviews. Pair programming and debugging will naturally go together. Interns can learn a lot by watching team members work through a problem, e.g., use of design patterns, test cases, and iterative development. We have our interns develop new functionality in a series of iterative steps, adding complexity as they show they’ve mastered the simpler parts of the problems.

## Rule 5: Choose tasks wisely

Start with small tasks to provide a sense of accomplishment. You’ll probably also want to start with noncritical tasks so that the rest of the team isn’t blocked. Use bug fixing as an opportunity for the intern to become familiar with existing functionality and design patterns. Fixing simple bugs can also provide a quick and instant reward for the intern. If possible, pick at least one task that will be in the upcoming release. We’ve seen faces just light up when they hear that something they’ve completed will soon be in the hands of paying customers; it’s a sense of accomplishment for them way beyond working on homework problems and course projects. Have a mix of bug fixing, new development, and exploratory tasks. Is there a new technology that your team hasn’t been able to spend time on yet? This can be a good opportunity to have an intern tackle that higher-risk project and provide added value to the company while being intellectually challenged. Allow for input from the interns; let them pick what they think they’d enjoy. We recently had a returning intern continue with a task from her previous summer. If the internship is of sufficient length, the intern can rotate through multiple teams or projects to get a broader view of the company’s products.

Set clear expectations for what the intern is to accomplish during his or her stay. Because interns are employed for only a short time, we use a simplified version of our objective setting and performance evaluation process. We develop fewer objectives for interns and typically ignore the usual weightings. By interacting so closely with the interns, we can skip the intermediate performance evaluation meeting. Our exit process on the last day includes the intern’s presentation, lunch, and a performance review.

## Rule 6: Help the intern learn about different parts of the organization

Many interns are surprised at how many other departments there are. We interact with, among others, quality assurance (QA), product managers, project managers, presales, sales, customer support, professional services, marketing, and information technology (IT). Learning about the other departments can improve an intern’s understanding of the overall commercial software industry. We typically schedule a number of 30- or 60-minute meetings so that our interns can learn and ask questions. This also exposes the interns to different career paths. And it’s an opportunity to meet people outside the development group. Encourage them to expand their network and connect on LinkedIn, Twitter, or other appropriate social media with the people they meet. Turning these meetings, in person or remote, into pizza lunches is a big hit.

## Rule 7: Interns should have fun

Make sure it’s not all work and no fun. This can be hard to keep in mind if an internship coincides with the end of a release cycle. Organize social activities with other interns. HR departments can be a big help here. Off-site events are always a favorite. This past summer, our interns spent an afternoon at a local escape room. Be sure to document (photograph/video) such events for the end-of-summer presentation (see Rule 9) or future intern marketing. Encourage team members to look for opportunities to involve interns. Ping pong and pool tables provide good break options. A warning sign is noticing that your intern is always eating lunch alone. Worse is having someone else point it out. Arrange lunches for your intern with other colleagues if need be.

## Rule 8: An intern is not a temp contractor

Make sure the intern leaves having learned something. Spend quality time with the intern; don’t assign tasks and then disappear. Provide opportunities to ask questions. Elicit questions. Students often don’t know what they don’t know. For them, problems that are hard, impossible, or unknown can all blur together. It can be hard to discern the difference and know how to start or continue. Ask leading questions to help an intern work through a problem without giving the answer. Remember that your organization’s, and your team’s, reputation will be shared with classmates when the intern returns to school. This can impact future hiring opportunities with that school.

## Rule 9: Host a "What I Did This Summer" presentation

It’s a great way to summarize what’s been learned and accomplished. The presentation can be used by the intern in classes or future interviews. We also use them to show new interns what their predecessors have done. Invite people outside of the immediate development team, e.g., research and development (R&D) leadership, HR, and people the interns have met or worked with (see Rule 6). These presentations are an excellent opportunity to communicate success to the rest of the organization.

## Rule 10: Get and give feedback

"So, how did we do? What can the company do better next summer?" Evaluate the intern as a possible future hire or a returning intern. Do you want them back? Do they want to come back? This is presumably one of the reasons your company has an internship program. Keep in touch with the intern. If the internship was a positive experience, offer to provide a letter of recommendation for future job and scholarship applications. Ask for intern candidates and new hire referrals. Find out when they’re back in town during school breaks. Most won’t say no to a free lunch with the team!

## References

[1] Aicher TP, Barabási DL, Harris BD, Nadig A, Williams KL (2017) Ten simple rules for getting the most out of a summer laboratory internship. PLoS Comput Biol [cited 2018 Nov 19];13(8): e1005606 Available from: 10.1371/journal.pcbi.1005606 28817622 PMC5560542

[2] Chen B (2014) Ten Simple Rules for Internship in a Pharmaceutical Company. PLoS Comput Biol [cited 2018 Nov 19];10(5): e1003600 Available from: 10.1371/journal.pcbi.1003600. 24811706 PMC4014388

[3] Masters KS, Kreeger PK (2017) Ten simple rules for developing a mentor–mentee expectations document. PLoS Comput Biol [cited 2018 Nov 19];13(9): e1005709 Available from: 10.1371/journal.pcbi.1005709. 28934208 PMC5608163

